# Translations on the Relations between the Medical and the Technical Portions of Dental Education

**Published:** 1912-03-15

**Authors:** Vincenzo Guerini

**Affiliations:** Naples


### TRANSLATIONS ON THE RELATIONS BETWEEN THE MEDICAL AND THE TECHNICAL PORTIONS OF DENTAL EDUCATION.

BY DR. VINCENZO GUERINI, NAPLES.

The subject to which I desire to draw your attention is perhaps one of the most important of the problems concerning the teaching of dentistry. Starting at the period when the first dental schools were established in America, there has always been more or less animated discussion as to the relations between the dental art and general medicine, and the extent to which medical knowledge must be included in the studies necessary to become a dentist. Even in our own days these discussions are not over. On the contrary, they have become more violent since the double qualification men have increased their numbers.

If we consider the organization of dental teaching in different countries we find enormous variety in this respect. There are some where predominating importance is given to technical instruction, as for example, in the United States and France; there are others in which medical education predominates, as in Austria and Italy, and again others, such as Germany, Switzerland and Belgium, where systems of organized dental teaching are in existence in each of which the relations between the dental portion and special general portion of the curriculum are differently regulated, but without giving too great influence either to the medical or the technical portion of study.

These differences are a source of continual discord which creates schism in the dental profession, diminishes its energies and prevents it from unanimously directing its efforts to one aim—the progress of the science and art of odontology.

We see continual changes taking place in the organization of dental studies in different countries, and these changes, as soon as they are made, excite keen criticism, and usually lead to further changes. Thus, in the United States the duration of studies which used to be three years was extended a short time ago to four years, and has since been again reduced to three years. A few years ago in Belgium a regulation was passed making it necessary to obtain the degree of candidat-medicine, before a student could present himself for the dental examination; but this arrangement is only transitory and it will be followed by further changes, the exact direction of which is not at present known, for great disagreement still reigns in Belgium as to the definite organization to be adopted for the teaching of dentistry.

In France dental teaching has just been reformed, with the result amongst other things, that the period of study has been lengthened from three to five years, but on reading the Ministerial report, which precedes the reform decree, there is clearly expressed there the idea that this reform also has a transitory character, and that perhaps in future the doctorate of medicine will be demanded of dentists.*

From the examples I have quoted it is clear that the greatest confusion still reigns on the subject of dental teaching and organization, and as to the organization it should have; the disagreement principally concerns the relation which must be maintained between the medical and dental portion of the student's education.

It seems to me, therefore, that it will be extremely useful to formulate a scheme of organization of dental teaching, which shall be capable of satisfying all demands and may serve as a guide in future reforms. One will thus be able to obtain reform which is inspired everywhere by the same fundamental principles, and which will have as a consequence the effect of gradually leading to uniformity of dental teaching in every country. This result could certainly not be obtained in a few years, but even if it were obtained in a century it would always be an immense advantage to the welfare of our profession.

The existence of the dental profession is threatened by the stomatological movement; at the present date this movement is rapidly increasing in Europe, and in America there are already a large number of partisans of this principle—that in order to be a dentist it is at first necessary to be a doctor.

---

*If the members of the commission dealing with the reform of medical studies is of opinion that the teaching of dentistry must logically be an integral part of the teaching of faculties of medicine, and if many consider that the diploma of doctor of medicine should be demanded of all dentists, it must not be forgotten that dental schools have their existence according to the law of 1875 on the ground of superior teaching, and only a new law could take away the prerogative. Consequently as things are at present, the only rational method consists in strengthening dental studies, and in surrounding the granting of the dental diploma with all the guarantees which experience has been able to suggest; besides raising the level of studies, is it not overcoming new obstacles and diminishing the distance which separates the two diplomas?

We must, therefore, defend ourselves against this invasion, which is intended to bring about the loss of the independence of the dental profession—this independence, which has been the principal cause of the wonderful progress in our art. I believe the best manner of defence is to organize the teaching of odontology in such an excellent manner that no reproach can be brought against it, and of even greater importance, that no charge of ignorance from the scientific point of view can be brought against dentists. It is well known that dentists have for a long time been treated with the utmost disdain; as their instruction has increased so they have acquired more esteem; it must be acknowledged that dental practitioners as a class are not appreciated in the way that medical men are; it is the same in every country. As a proof of it we have only to refer to the fact that in America many dentists, after having obtained the degree of doctor of dental surgery, continue their studies in order to obtain that of doctor of medicine, for the latter secures for them a greater degree of consideration and prestige. Dentistry, then, must be freed from this position of inferiority; the dentist must equal the doctor from every point of view. To make this possible it is necessary that the general and scientific culture of the dentist shall be raised to the same standard as that of the medical man, which is not only necessary for social amelioration but also in the interest of the scientific progress of our profession.

Firstly, it is necessary that the preliminary studies demanded for admission to dental schools shall be the same as those required for admission to medical schools. In 1904 the International Dental Federation established this principle, but it is to be regretted that it has not been universally adopted.

Let us consider what part the teaching of natural and medical sciences must take in the instruction of dental students. At Stockholm the International Dental Federation ruled that scientific and medical studies of dental students must include the following subjects:

1. Physics.
2. Chemistry, including metallurgy.
3. Anatomy.
4. Histology and embryology.
5. Physiology, including chemical physiology.
6. Bacteriology.
7. Materia medica and therapeutics.
8. General pathology.
9. General surgery.
10. Diagnosis.
11. Special surgery and anesthesia.

This programme of scientific and medical studies has been recognized as indispensable by the International Dental Federation in order to turn out well-trained dentists who would be able to give an exact account of pathological phenomena in relation to the teeth and to practise their profession, not in an empirical manner, but on the solid basis of scientific knowledge. It is therefore greatly to be regretted that the programme of medical studies so wisely proposed has not yet been consistently adopted. Even in France, where the duration of studies has been extended to five years, no heed has been paid to this programme.

The medical studies prescribed by the new regulations are quite rudimentary and among other things they do not include certain subjects of fundamental importance, such as general pathology and general surgery.

You know very well that the pathological processes which take place in relation to the teeth, the gums, the jaws and adjacent tissues are processes identical in their nature with those seen in other parts of the body. In the dento-maxillary regions we meet, as elsewhere, anaemia, hyperaemia, inflammation, suppuration, gangrene, benign and malignant neoplasms, etc., etc. It is therefore absolutely impossible to understand dental pathology thoroughly without having studied all these morbid processes, their cause and termination, as well as the anatomical and histological alterations which characterize them and result from them.

That is to say, the scientific study of dentistry demands as an indispensable condition, a knowledge of general pathology and general pathological anatomy, which is the study of all the different kinds of morbid processes in their widest aspect.

On the other hand it is absolutely impossible to understand general pathological processes without knowing the anatomical and histological constitution of the body and the functions of the organs, in other words, without a good groundwork in anatomy, histology and physiology.

These subjects are of fundamental importance; it is necessary that they should be studied thoroughly, for there is nothing worse than superficial scientific knowledge; it engenders imperfect and confused ideas which are often erroneous, and real ignorance is only hidden by simulation of knowledge. Good dental teaching must have as its object the turning out of dentists who are not only clever as operators and prosthetists, but who shall have a thorough knowledge of the teeth and neighbouring tissues, in addition to the relations between dental diseases and those of the rest of the body. These relations are very numerous and complex, and they must be known in order that the etiology and prevention of dental disease may be understood.

It is, therefore, necessary that dental teaching shall be seriously scientific in character and must include the entire general portion of the medical students' training.

To make my point very clear, allow me to point out to you that medicine is at the same time a science and an art; and that in consequence medical studies include a scientific portion and a practical portion; the scientific portion embraces specially the studies of anatomy, embryology, physiology, etc., etc., The practical portion includes chiefly instruction in the various clinics, *e. g.*, the surgical clinic, as well as special clinics, such as skin and eyes.

Now this portion of medical education, that is the special or practical portion, is almost entirely useless to dentists, but the general or scientific portion is as necessary to the dental student as it is to the medical student, and therefore the former must devote as much time and work to them as the latter. If, now, we ask how much time must be devoted, in the teaching of dentistry, to general scientific subjects, the reply can only be this—the same length of time as medical students devote to these studies. Being anxious to state as precisely as possible this amount of time, I have secured information on the subject from the medical schools of Italy and other countries. From the figures which have been communicated to me I have reckoned that the total duration of medical teaching calculated in hours varies between 4,000 and 6,000 and that about half of this time is devoted to general scientific teaching, whilst the other half is given over to the different clinics, special pathology, operations, jurisprudence, public health, and similar subjects, instruction in which is not necessary for the dentist. We see, then, that in the course of medical study the total time devoted to the teaching of general scientific subjects, that is subjects the study of which is necessary for dentists, is from 2,000 to 3,000 hours. We must, therefore, come to the conclusion that if we wish to give dentists a scientific medical education, sufficient in all respects, it is necessary to allot to this teaching at least 2,000 hours.

Let us compare this amount of time with the minimum time which is regarded as necessary for teaching purely dental subjects. The F. D. I. resolved that doctors desiring to practice dentistry should devote to the study of the specialty at least two school years of 1,200 hours each; that means that the minimum time considered necessary by the F. D. I. for teaching special dental subjects is 2,400 hours, but, since it would be prejudicial to contract the technical and practical teaching of dentistry into too short a time, and since, in the best dental schools, especially in America, the total time devoted to special teaching is not less than 3,000 hours, we may consider this figure as the minimum time which it is necessary to devote to the special teaching of dentistry. To conclude, then, in order that the teaching of odontology may be as complete on the scientific as on the practical side, it is necessary to devote at least 2,000 hours to the scientific medical subjects and at least 3,000 hours to purely dental subjects which brings up the total number of hours for the dental curriculum to at least 5,000. A very important practical deduction follows as a consequence, if we wish to have dental teaching thoroughly replete—*the curriculum must extend over at least four or five years.* A very simple calculation gives us this result: the number of hours in a school year never exceeds 1,200 to 1,300; usually it is about 1,000 hours, and since the total duration of dental teaching must not be less than 5,000 hours, it is obvious that with school years of 1,200 to 1,300 hours each, four school years will be necessary to obtain the 5,000, whilst with school years of 1,000 hours each five years would be necessary to obtain this total of time. It is, therefore, necessary to choose between a four year and a five year course. Teaching divided into four school years would have the advantage of taking up one year less, but has the great disadvantage that students must spend in the school seven or eight hours daily,* and so very little time remains for them to study at home, a *sine qua non,* if the scientific information they have learned in the schools is to be fixed in their minds.

On the other hand, the five year course has the double advantage of being organized in a much more careful and complete manner, and of leaving the students more time to study in their own homes in order that they may assimilate better the gist of the teaching, especially as regards scientific studies.—*Dental Record.*

---

*The number of teaching days in a school year after the deduction of the vacation holidays and examination periods varies between 150 and 180, and that even in America where they are so assiduous and laborious. That being so, it is very easy to calculate that seven to eight hours teaching per day are necessary to reach a total of 5,000 hours' instruction in four years.
TRANSACTIONS INTERNATIONAL DENTAL CONGRESS.

